# Benefits of Taking a Virtual Field Trip in Immersive Virtual Reality: Evidence for the Immersion Principle in Multimedia Learning

**DOI:** 10.1007/s10648-022-09675-4

**Published:** 2022-04-22

**Authors:** Guido Makransky, Richard E. Mayer

**Affiliations:** 1 Department of Psychology, University of Copenhagen, Øster Farimagsgade 2A, 1353 København K, Copenhagen Denmark; 2 Psychological and Brain Sciences, University of California Santa Barbara, Psychology East, Room 3841, Santa Barbara, CA 93196-9660 USA

**Keywords:** Immersion, Virtual reality, Video, Virtual field trip, Affective processing, Head mounted display, Metaverse

## Abstract

This study describes and investigates the immersion principle in multimedia learning. A sample of 102 middle school students took a virtual field trip to Greenland via a head mounted display (HMD) or a 2D video as an introductory lesson within a 6-lesson inquiry-based climate change intervention. The HMD group scored significantly higher than the video group on presence (*d* = 1.43), enjoyment (*d* = 1.10), interest (*d* = .57), and retention in an immediate (*d* = .61) and delayed posttest (*d* = .70). A structural equation model indicated that enjoyment mediated the pathway from instructional media to immediate posttest, and interest mediated the pathway from instructional media to delayed posttest score, indicating that these factors may play different roles in the learning process with immersive media. This work contributes to the cognitive affective model of immersive learning, and suggests that immersive lessons can have positive longitudinal effects for learning.

## Introduction

Ana, a middle school geography teacher is in the process of deciding on how she will teach her class about climate change, and is considering using a nationally available inquiry based science learning (IBSL) intervention that lasts six lessons. In this intervention, students are assigned to groups and play the role of a research team that has to virtually travel to Greenland to investigate the evidence and consequences of climate change. They are then required to develop a number of experiments, and then present their results to a hypothetical UN panel of experts. The intervention is available in two versions where students can either experience climate change in Greenland with a 3D video presented via a head mounted display (3D HMD; i.e., a higher-immersion medium) or by watching the same content as a 2D video viewed on a projected screen (i.e., a lower-immersion medium). Ana’s school has recently purchased HMDs, but she is unsure of the potential added learning and motivational value of using them compared to the more conventional video solution.

Ana’s dilemma is one that many educational stakeholders will be facing in the coming years. Although there is abundant literature on 2D virtual field trips in education (e.g., Spicer & Stratford, 2001; Tuthill & Klemm, 2002), and studies that investigate the value of 3D HMD based virtual field trips (Markowitz et al., 2018; Petersen et al., 2020), fewer studies have systematically investigated the educational value of using a 3D HDM instead of a 2D video, for presenting a virtual field trip in a real educational setting. This article provides an overview of the immersion principle in multimedia learning that builds on existing evidence of the value of immersive learning experiences in education. Furthermore, it describes an experiment to test the immersion principle in the above mentioned IBSL intervention in a real middle school education context, with the purpose of uncovering evidence concerning the value of integrating immersive media into educational interventions.

It is useful to distinguish between *immersion*—which involves objective features of the instructional technology—and *presence*—which involves the learner's subjective experience. In particular, immersion is described as an objective measure of the extent to which a system presents a vivid virtual environment while shutting out physical reality (Cummings & Bailenson, 2016; Slater & Wilbur, 1997), whereas presence is the psychological sense of ‘being there’ in the environment depicted by the virtual simulation (Slater, 1999). In the present study, we vary immersion by comparing learning with a 2D projection on a screen versus a 3D HMD system with a head mounted display, and we measure presence through a self-report survey.

## Objective

The immersion principle in multimedia learning describes how immersive virtual environments promote better learning when they incorporate multimedia design principles (Makransky, n.d.; Mayer, 2021a). That is, the immersion principle in multimedia learning predicts that people learn better with immersive media (e.g., a 3D video experienced through a HMD) than with less immersive media (e.g., equivalent instructional video presented on a 2D screen; Makransky, n.d.; Mayer, 2021a), when immersive lessons are designed according to instructional design principles and the affordances of the media (Makransky & Petersen, 2021). In short, we are interested in two main questions about immersion as an instructional variable: does it work and how and why does it work.

Concerning the issue of does immersion work, our primary research question is: Do students learn better when content is presented in a higher-immersion medium than a lower-immersion medium? To answer this question, we employ a media comparison experiment (Clark, 2001; Mayer, 2014) in which we compare the learning outcomes (and ratings of presence, interest, and enjoyment) of students who learn about environmental science in an immersive 3D video experienced through a HMD (i.e., higher-immersion medium) or through a 2D video viewed on a projected screen in the front of the classroom (i.e., lower-immersion medium). Concerning the issue of how does immersion work, our research question is: Do presence, interest, and enjoyment mediate the effects of immersion on test performance? To answer this question, we build on the Cognitive Affective Model of Immersive Learning (CAMIL; Makransky & Petersen, 2021) and use structural equation modeling (SEM; Kline, 2011) to test a model of learning with immersive media.

In the following sections, we will introduce readers to the use of immersive virtual reality (IVR) in education and their previous use in climate change education, followed by an introduction to the immersion principle in multimedia learning. Then, we will present the state of the art and gaps in the literature in the field of immersive learning, which motivate five hypotheses about if, and how, the immersion principle in multimedia learning works in the IBSL middle school intervention summarized above.

## Immersive Virtual Reality in Education

With the increased quality and availability of affordable IVR systems involving head-mounted displays (HMDs), the number of regular IVR users in the UA. increased from 30.6 million to 45.3 million from 2018 to 2019, and were projected to be 55.3 million in 2020 (Artillery Intelligence, 2020). This upward trend is expected to continue following the global pandemic which has forced many educational institutions to restructure their educational initiatives and search for online alternatives to traditional teaching methods (Remtulla, 2020; Singh et al., 2020), and the expectation that the Metaverse could be the next iteration of the internet (Pimentel et al., in press). Furthermore, education is the industry where the use of IVR is growing most rapidly, and is an industry where demand greatly exceeds supply (Superdata, 2020). The number of research studies that investigate the use of VR in education is also rapidly increasing and a search on the Scopus database with the search terms virtual reality and either education, teach, learn, or train identified 2477 articles from 2020, compared to 1143 in 2015, and 869 from 2010. While the number of studies related to using VR in education is rapidly increasing, recent reviews highlight a general lack of theoretical and methodological rigor in most of the studies in this field. Criticisms include, the lack of learning theories and best practices to guide IVR application development (Di Natale et al., 2020; Radianti et al., 2020), non-validated measures (Di Natale et al., 2020), the lack of high quality experimental studies (Jensen & Konradsen, 2018), the lack of integration within actual teaching or learning interventions (Radianti et al., 2020), and the need for longitudinal studies (Mikropoulos & Natsis, 2011).

## Using IVR in Climate Change Education

A systematic literature review investigated the use of various immersive virtual environments (including augmented, mixed, and virtual reality), identified an increasing number of studies on the topic of climate change (Queiroz et al., 2018). In a recent review of the field, Fauville et al., (2020) categorized these studies according the three components of engagement necessary to elicit change in the public perspective of climate change, proposed by Ockwell et al. (2009) including: understanding, emotion, and action. Several studies have investigated the value of IVR for promoting the understanding dimension, which is the dimension that is relevant for the present study. Moreno and Mayer (2002) investigated the impact of different media and instructional methods in a virtual botany learning activity and did not find any differences across more and less immersive media conditions. Using the same virtual botany learning activity Moreno and Mayer (2004), investigated the impact of personalized message on learning in low and high immersion conditions. Students reported higher levels of physical presence in the high immersion condition but this did not lead to better performance on tests of retention or transfer.

In a more recent study Markowitz et al. (2018) used several experiments to test the efficiency of IVR as an educational medium for teaching the consequences of climate change, focusing on the topic of ocean acidification with positive results. Finally, Petersen et al., (2020) investigated the effect of the pre-training principle in HMD based climate change intervention. The results revealed that students in both conditions had significant increases in declarative knowledge, self-efficacy, interest, STEM intentions, outcome expectations, and behavioral change intentions. They also found a significant difference between conditions on the transfer test favoring the pre-training condition. In general, a clear gap in this literature is the need to specifically investigate if there is added educational value when presenting climate change lessons with high-immersion media (e.g., HMDs) compared to less immersive media. In this paper, we present the immersion principle in multimedia learning, and test it in a longitudinal study that takes place in a middle school context using the IBSL intervention on climate change that was introduced above.

## The Immersion Principle in Multimedia Learning

The immersion principle in multimedia learning proposes that immersive virtual environments promote better learning when they incorporate multimedia design principles (Makransky, n.d.; Mayer, 2021a). The immersion principle holds that immersive media per se do not necessarily improve learning; however, implementing effective instructional methods within immersive virtual environments or contextualizing immersive learning experiences within a lesson can improve learning (Makransky, n.d.). The goal of effective instructional design or contextualizing immersive virtual environments within lessons is to promote the learner's cognitive processes of selecting, organizing, and integrating information (Makransky et al., 2021). The instructional design principles or generative learning strategies that are specifically effective for learning in IVR, either facilitate the features that make immersive learning environments special (i.e., the affordances; Bailenson, 2018; Bailenson et al., 2008; Makransky & Petersen, 2021), or mitigate the limitations related to learning with immersive technology (Baceviciute et al., 2021; Makransky, n.d.; Parong & Mayer, 2020).

Research has identified several affordances of immersive virtual learning environments. One is the ability to immerse students in virtual environments that they can explore in first person, but may be impossible, too expensive, dangerous, or impractical to explore in the real world (Bailenson, 2018). This provides unique experiential and situated learning opportunities that are not normally accessible (Di Natale et al, 2020). Another is the possibility to contextualize learning through highly realistic scenarios that can support situated learning and enhance transfer of learning (Di Natale et al., 2020). Finally, they provide engaging experiences that promote motivation (Di Natale et al., 2020).

Learning is thus immersive based on the extent that the visual, auditory, and haptic cues are from the virtual environment rather than physical reality. Systems can be more or less immersive based on technological factors such as the tracking level, stereoscopic vision, image and sound quality, field of view, and update rate. Di Natale et al. (2020) differentiate between non-immersive systems such as desktop VR, semi-immersive systems such as a full dome or smart glasses, and fully immersive systems such as HMDs. According to the CAMIL (Makransky & Petersen, 2021), the main affordances of learning in immersive environments are a higher sense of presence (the psychological sense of ‘being there’) and agency (being in control of one’s actions). Furthermore, cognitive load has been identified as a potential limitation when learning with IVR (Makransky et al., 2019b; Parong & Mayer, 2020). Therefore, more immersive environments will specifically benefit lessons that rely on the affordances of presence or agency and instructional designers and educators should be aware of the specific affordances and limitations of the using immersive technology.

The immersion principle of multimedia learning builds on theories of interest (e.g., Renninger & Hidi, 2016), motivation (e.g., Deci & Ryan, 2000), and multimedia learning (e.g., Mayer, 2014) to describe how more presence can lead to better learning outcomes when this affordance is constructively used in designing a lesson. For instance, social agency theory suggests that a higher presence can lead students to engage in deeper cognitive processing and investing more cognitive effort to understand material (Mayer, 2014). The immersion principle of multimedia learning would therefore predict that learning would be improved when the instructional intervention depends on a high sense of presence, such as a scenario where students virtually travel to Greenland to experience the consequences of climate change. In this case, the experiential learning opportunity is appropriate for IVR because it is too expensive in the real world. Furthermore, experiencing the virtual field trip as real (i.e., higher presence) could lead students to engage in deeper cognitive processing, and this would lead to more learning as long as the lesson is designed so that presence helps students focus their attention on selecting, organizing, and integrating relevant learning content, rather than distracting them from this content (Makransky et al., 2019b) .

## Evidence Related to the Immersion Principle in Multimedia Learning

A number of reviews and a meta-analysis have recently investigated the value of using immersive virtual lessons with different aims. Jensen and Konradsen (2018) identified 21 studies of immersive learning and training through HMDs. They highlighted situations where immersion is useful, including skill acquisition related to remembering and understanding spatial and visual information and knowledge; psychomotor skills related to visual scanning or observational skills; and affective skills related to controlling emotional response to stressful or difficult situations. They did not identify advantages when compared to less immersive technologies outside of the above mentioned areas, and noted that immersion could be counterproductive in some cases because of factors such as cybersickness, technological challenges, or when the immersive experience distracted learners from the learning task.

Radianti et al. (2020) conducted a systematic review of IVR applications in higher education and identified 38 studies. Their review describes 18 promising application domains, and propose a research agenda, but they do not make any conclusions regarding learning outcomes. A recent meta-analysis by Wu and colleagues (2020) synthesized the findings from 35 studies comparing immersive VR to less immersive desktop VR and other traditional means of instruction. They found an advantage in favor of more immersive media and suggest that using HMDs can improve knowledge acquisition as well as skill development. Although not included in the meta-analysis, the results from research investigating the affective outcomes of the immersion principle in multimedia learning are fairly consistent showing that more immersive environments yield higher presence (e.g., Makransky & Lilleholt, 2018; Makransky et al., 2019b), enjoyment (e.g., Makransky & Klingenberg, 2022; Makransky et al., 2019a; Meyer et al., 2019), and interest (e.g., Makransky et al., 2020).

Although the meta-analysis included several moderator variables, it did not consider the factor of instructional design. Several studies suggest that incorporating instructional design and scaffolding principles from less immersive media may result in greater learning outcomes in immersive media. These include the pre-training principle (Petersen et al., 2020), the segmentation principle (Parong & Mayer, 2018), the personalization principle (Makransky et al., 2019c; Moreno & Mayer, 2004), the signaling principle (Albus et al., 2021), and the modality principle (Moreno & Mayer, 2002). In some cases, instructional and scaffolding strategies have also been found to be more effective in highly immersive environments including pre-training (Meyer et al., 2019), and the generative strategies of summarizing (Klingenberg et al., 2020), and enacting (Makransky et al., 2021). Therefore, higher immersion may be more effective when appropriate instructional design and scaffolding approaches are used (Parong & Mayer, 2018).

Finally, previous studies have investigated the immersion principle of multimedia learning by comparing lessons presented in fully immersive systems such as HMD’s to the same lesson presented in a less immersive system such as desktop VR or video (e.g., Buttussi & Chittaro, 2018; Checa and Bustillo, 2020; Krokos et al., 2019; Makransky et al., 2019a; Makransky et al., 2019b). By doing so researchers attempt to isolate the effect of immersion on motivational and learning outcomes. Since immersion is the extent to which a system shuts out sensations from the ‘real world’, accommodates many sensory modalities, and has rich representational capability (Slater & Wilbur, 1997), a lesson experienced through a HMD is regarded as more immersive than a lesson experienced through a 2D video. One objective of more immersive systems is to produce realistic experiences (Bowman & McMahan, 2007). While other factors such as interaction and agency (Johnson-Glenberg, 2019; Makransky & Petersen, 2021; Petersen et al., 2022) can play a role when investigating the immersion principle in multimedia learning, the major psychological difference between learning in a more and less immersive environment is the level of psychological presence (Johnson-Glenberg, 2019; Makransky et al., 2021).

## How Immersion Influences Learning: a Cognitive Affective Model of Immersive Learning and Hypotheses

In the current study we wish to add to the literature on the immersion principle in multimedia learning by investigating the short-term (immediately after the virtual field trip) and long-term (an average of approximately 3 weeks after the field trip) impact of using either a 3D video accessed through a HMD or 2D video to provide a virtual field trip as an early experience within a larger inquiry-based module on climate change. In addition, we investigate how affective factors including presence, enjoyment, and interest are involved in the learning process using structural equation modeling (SEM).

**Fig. 1:**
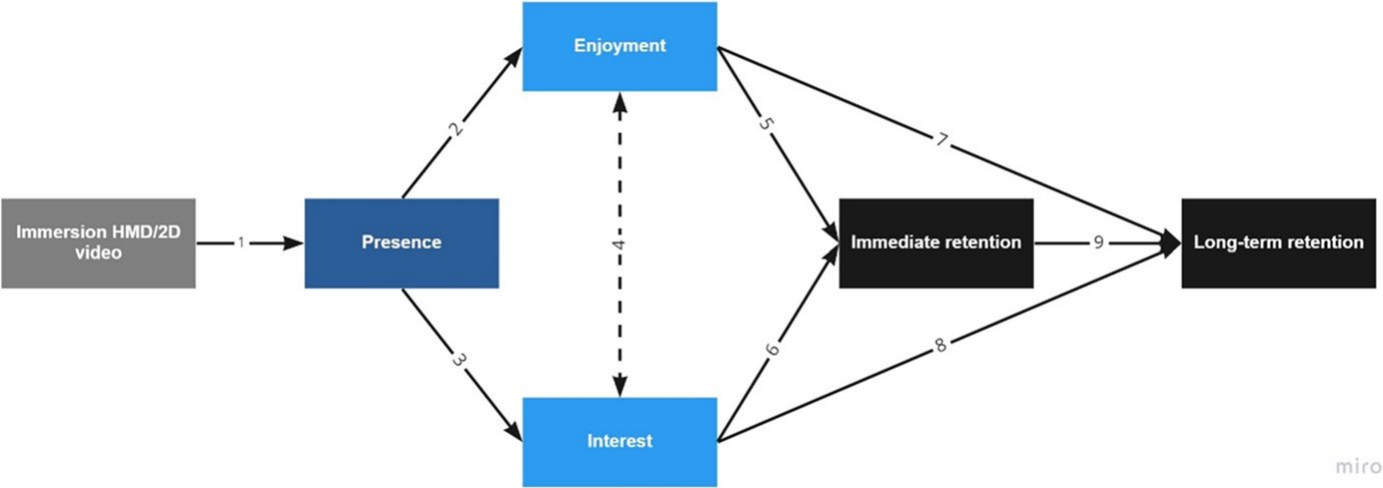
Hypothesized relationships based on the cognitive affective model of immersive learning

Figure 1 presents a cognitive affective model of immersive learning that uses existing literature to describe how level of immersion may influence learning. The model outlines how immersion (HMD vs. video) influences presence (link 1). Presence further is related to the affective factors of enjoyment (link 2) and interest (link 3). Enjoyment is also expected to co-vary with interest (link 4). Enjoyment is further be related to immediate retention (link 5) and long-term retention (link 7), and interest is related to immediate retention (link 6) and long-term retention (link 8). Finally, immediate retention is related to long-term retention (link 9). The first link in the model is between immersion and presence, which has been supported in previous literature (Cummings & Bailenson, 2016). In a meta-analysis, Cummings and Bailenson (2016) found that level of immersion has a medium-sized effect on presence. They also found that increased levels of user-tracking, the use of stereoscopic visuals, and wider fields of view of visual displays were the most important immersive system features for developing presence. Therefore, Hypothesis 1 in this study is: Students in the 3D HMD group will report a higher level of presence than students in the 2D video group.

The second link in the model presented in Fig. 1 is between presence and enjoyment. Makransky and Lilleholt (2018) used SEM to investigate how immersion may prime affective and cognitive process in the learner that support motivational and learning outcomes. They identified two paths through which higher immersion leads to perceived learning outcomes: an affective path and a cognitive path. The affective path describes how immersive learning experiences can facilitate presence which in turn facilitates positive affective outcomes such as enjoyment and motivation. Highly immersive lessons can be experienced as more enjoyable than less immersive lessons (Makransky & Lilleholt, 2018; Meyer et al., 2019), because learners feel like they are part of a high fidelity virtual environment wherein they have meaningful social interactions. Therefore, Hypothesis 2 is: Students in the 3D HMD group will report that they enjoy the virtual field trip more than students in the 2D video group.

Regarding link 3, presence can also spark situational interest in the learner (Parong & Mayer, 2018), through novel and intense learning experiences (Hidi & Renninger, 2006; Renninger et al., 2008). According to Renninger and Hidi's (2016) four stage theory of interest, situational interest is a kind of interest that is caused by exciting and enjoyable features of the instructional episode. In the present study, we are particularly interested in whether students who learn in a higher-immersion environment report higher levels of interest, and the degree to which there is a relation between presence and interest. This link is supported in a recent research which found that an IVR simulation resulted in higher presence and interest compared to the same lesson presented by video (Makransky et al., 2020; Petersen et al., 2022). Therefore, Hypothesis 3 is: Students in the 3D HMD group will report higher interest after experiencing the virtual field trip compared to students in the 2D video group.

Link 4 in Fig. 1 takes into account the theoretical relation between enjoyment and interest. While interest motivates exploration of what is novel and intriguing, enjoyment is the sense of satisfaction and reward generated from the activity and/or the outcome of the activity (Ainley & Hidi, 2014). Tomkins (1962) proposed that the relation between these two variables is reciprocal, which is represented through a non-directional relationship in Fig. 1. Although there is typically overlap between enjoyment and interest, and interest-based actions are often associated with positive emotional experiences (Hidi & Harackiewicz, 2000), this is not always the case. An example is that medical students may find dissecting cadavers to be interesting but may simultaneously experience negative affect related to the experience. Renninger (2000) describes how enjoyment associated with achievement of a solution to a problem may only be experienced after persisting through earlier frustration. Finally, evidence suggests that interest and enjoyment may be triggered separately at different points as students undertake new and complex learning tasks, which highlights the need to look beyond global measures of positive emotion (Ainley & Hidi, 2014). Therefore, the next links (5–8) distinguish between the how enjoyment and interest relate to measures of learning outcome.

Interest theories of motivation (Dewey, 1913; Renninger & Hidi, 2016) posit that when students find interest in the material they may learn more deeply and therefore perform better on measures of learning outcome. Enjoyment emotions benefit performance by focusing attention on the task itself, and can lead to higher intrinsic motivation (Pekrun, 2006), and higher levels of generative processing (Makransky et al., 2019a; Petersen et al., 2022). Therefore, enjoyment and interest are both related to learning, although they can be triggered separately at different points as students undertake complex learning tasks (Ainley & Hidi, 2014). This leads us to Hypothesis 4 which is: Students who experience the virtual trip via a HMD will perform better than students who experience the virtual trip as a 2D video on an immediate posttest.

Several studies suggest that highly immersive lessons lead to favorable learning and motivational outcomes compared to less immersive lessons when proper scaffolding strategies are introduced prior to or after the immersive lesson. These include the use of pre-training prior to a lesson (Meyer et al., 2019), or the inclusion of generative learning strategies after a lesson (Klingenberg et al., 2020; Makransky et al., 2021). In the current study, the virtual field trip was integrated in the exploration phase of an IBSL intervention (Stainfield et al., 2000). This was followed by four lessons where students could apply what they had learned in the virtual lesson, thereby ensuring that students had considerable reflection opportunities regarding their virtual visit to Greenland.

Several studies have highlighted the possibility that assessing the short-term impact of leaning with IVR based lessons may not be capturing the actual value of using immersive technology (e.g., Makransky et al., 2019a, 2020). This is specifically the case because one major affordance of learning with immersive media is a high level of interest, enjoyment, and engagement, resulting in higher intrinsic motivation (Makransky & Petersen, 2019). This could lead students to exert more effort and ultimately more time on task, which could eventually result in better learning, which is not adequately captured in a short-term assessment (Makransky, Borre-Gude, et al., 2019). This leads us to Hypothesis 5 which is: Students who experience the virtual trip in HMD will perform better than students who experience the virtual trip as a 2D video on a delayed posttest that takes place an average of 19.44 days (*SD* = 9.87) after the virtual field trip.

While the affective path typically facilitates learning through higher generative processing, there are also boundary conditions. Cognitive factors such as cognitive load and reflective thinking can either impair or benefit learning depending on the instructional design of the immersive lesson (Makransky & Petersen, 2019). Cognitive load theory (CLT; Sweller et al., 2011) and the cognitive theory of multimedia learning (CTML; Mayer, 2014, 2021a, b) describe how cognitive overload occurs if the information to be processed during learning exceeds the limited capacity of working memory. While immersive environments can diminish extraneous cognitive load by shutting out potential distractions from the physical environment (Baceviciute et al., 2020), research also shows that immersive environments can increase cognitive load because learners have to relate to a greater visual field of view, which typically increases complexity (Makransky et al., 2021). A higher level of representational fidelity can also interfere with the sense-making process when seductive details that are not necessary for learning are included (Moreno & Mayer, 2002). Extraneous factors can also tempt learners to engage in hedonic activities that are not beneficial to learning but may have entertainment value (van der Heijden, 2004). Therefore, although the recent meta-analysis by Wu et al., (2020) highlights the benefits of learning in more immersive environments, several studies have found that this is not always the case (e.g., Johnson-Glenberg et al., 2020; Parong & Mayer, 2018), and some studies specifically highlight additional extraneous cognitive load caused by high immersion as an explanation (Makransky et al., 2019b; Meyer et al., 2019; Parong & Mayer, 2018, 2020).

Another boundary condition identified in the literature on the immersion principle of multimedia learning is related to reflection and self-regulated learning (Zimmerman, 2013; Zimmerman and Schunk, 2001). Immersive learning environments can facilitate social presence in learners, making it possible to increase reflection and self-regulated learning through meaningful interactions with pedagogical agents or peer avatars (Makransky et al., 2019c). Nonetheless, immersive learning environments are highly engaging, yet cognitively demanding, so reflection can suffer when immersive lessons do not provide natural reflection opportunities (Makransky et al., 2019b). This is the case because learners are often highly stimulated, but this may hinder self-regulated learning as the learner may not actively monitor or adapt their affective, cognitive, metacognitive, and motivational processes unless lessons are heavily scaffolded (Makransky et al., 2021; Meyer et al., 2019; Parong & Mayer, 2018). Furthermore, many educational VR lessons are not well designed and lack strong pedagogy, as noted by Johnson-Glenberg (2019).

In summary based on the immersion principle in multimedia learning we wish to explore possible pathways between the level of immersion during instruction and scores on a long-term test of learning outcome in order to better understand the role of affective factors such as presence, interest, and enjoyment in the mechanism of change. In particular, we test the foregoing five hypotheses in this study.

## Method

### Participants and Design

The participants were 102 students between the ages of 13 and 16 (*M* = 14.14, SD = 0.675). The students were either in 8^th^ grade (*n* = 82) or 9^th^ grade (*n* = 20) and were from four different public schools from different regions in a European country. The students reported their gender as 39 boys, 63 girls, and 0 non-binary. A sensitivity power analysis was conducted to estimate the minimum effect size detectable for an independent samples t-test between two groups (*n* = 49, *n* = 53) with 80% power, and *α* = 0.05 using G*Power 3.1 (Faul et al., 2007). The analysis revealed that a medium effect size (Cohen's *d* = 0.50) would be detectable with the current sample size. In a between-subjects experimental design, 49 students served in the HMD group (in which they took a virtual field trip accessed through an 3D HMD) and 53 served in the 2D video group (in which they took a virtual field trip with 2D video projected on a screen in front of the classroom). The study was conducted in a European country with all materials in the local language.

### Materials

The measurement materials consisted of a prequestionnaire soliciting demographic information (age, grade, and gender), an immediate posttest consisting of presence, interest, and enjoyment surveys and a knowledge test, and a delayed test consisting of the same knowledge items as in the immediate posttest. The presence scale consisted of five items adapted from the physical presence sub-scale in Makransky et al. (2017). Three of the items were worded exactly as in the original scale (e.g., “I was completely captivated by the virtual world”); however, two items were reworded slightly in order to make them more specific to the current lesson thereby limiting ambiguity (e.g., “The virtual environment seemed real to me” was changed to “The virtual field trip to Greenland seemed real to me”). The interest scale consisted of four items adapted from Thisgaard and Makransky (2017; e.g., “I am interested in the scientific explanations for climate change”). The enjoyment scale consisted of two items adapted from Tokel and İsler (2015; e.g., “I like to learn about climate change through VR/video”). The presence, interest, and enjoyment scales used a five point Likert scale. The knowledge test consisted of seven multiple-choice and four polytomous items designed to measure knowledge about concepts such as the greenhouse effect and albedo effect (see Appendix Table 2 for the full list of items). The items were developed by a team consisting of teachers, educational psychologists, and psychometricians with the goal of having a short measure that would be able to assess students' general knowledge of the learning material.

**Fig. 2:**
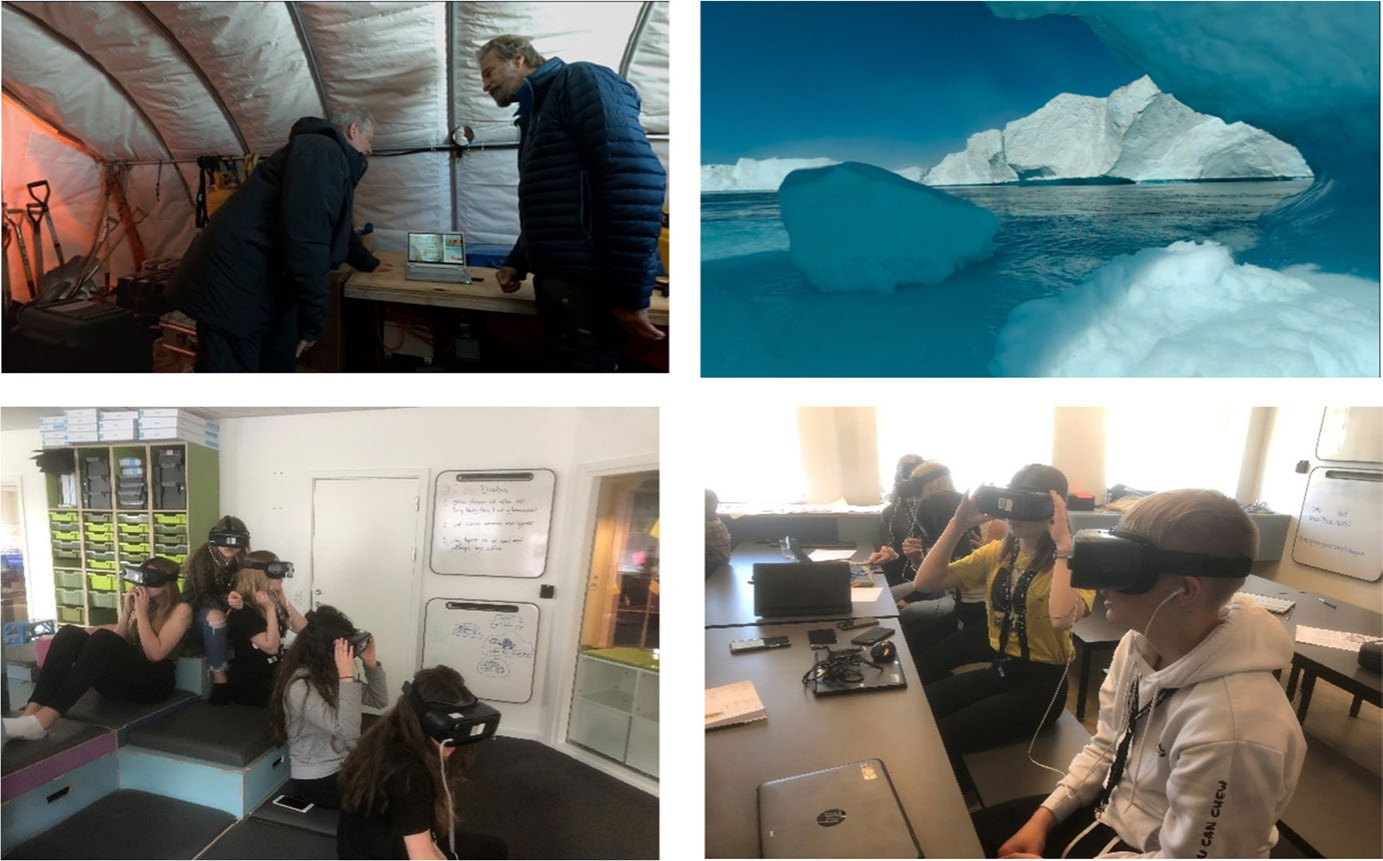
Screen shots of the virtual field trip to Greenland in the top panels, and picture of students using HMD virtual field trip in the lower panels

The instructional materials consisted of a 3D HMD version or a 2D video version of a virtual field trip to Greenland to experience the consequences of climate change. The virtual field trip was built on a documentary by Dennis and Strauss (2018) labeled, *This is Climate Change: Melting Ice*. Students began the virtual field in a helicopter which lands at a science base camp in Greenland, where they then follow former US Vice President Al Gore who was visiting a scientist at the base. Students were able to experience the consequences of the changing climate from a first person perspectives as shown in the top panels of Fig. 2. Students observed the extraordinary amounts of melting ice while listening to the scientist explain the current situation compared to past measurements. Because the learning content in the virtual field trip was limited, a narration was recorded and merged to the existing audio using professional audio equipment (Yeti from Blue). This made it possible to deliver necessary learning content based on instructional design principles including the multimedia principle (Mayer, 2021b), signaling principle (Albus et al., 2021; van Gog, 2021), split attention principle (Ayres & Sweller, 2021), redundancy principle (Baceviciute et al., 2022; Kalyuga & Sweller, 2021), modality principle (Baceviciute et al., 2020; Castro-Alonso and Sweller, (n.d.)), and personalization principle (Fiorella & Mayer, 2021b; Petersen et al., 2021). The virtual environment provided a contextualization; however, the recorded narration contained all of the necessary information for answering the post-test questions. The virtual field trip thus contained important declarative knowledge about climate change which they needed as the foundation for the remaining learning activities in subsequent lessons.

### Apparatus

All students experienced the same educational content, with the only difference being the viewing device. The students in the 3D HMD group experienced the virtual field trip as a 360° video administered through Samsung S7 or S8 phones using the Samsung Gear VR head mounted display as shown in the bottom panels of Fig. 2. Students could change their point of view (POV) and look around the 360° 3-dimensional environment as on a virtual tour, however there was no navigation beyond gaze. The HMD features rotational tracking, but no positional tracking. Hence, head movement was used to change the participant's field of view and dynamically render the 360° virtual space. In contrast, students in the video condition experienced the virtual field trip as a 2D video projected onto a large screen in the classroom. The virtual field trip lasted for 9 min and 46 s.

### Procedure

**Fig. 3:**
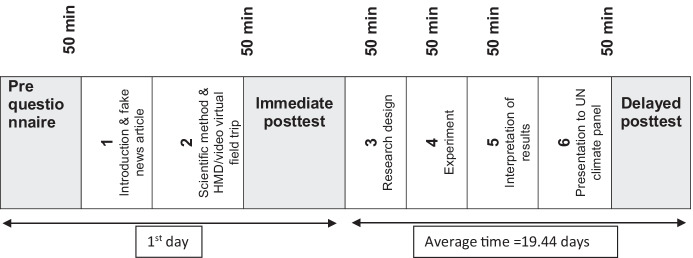
Illustration of the six lesson within the IBSL learning intervention about climate change

The virtual field trip was an integrated part of a nationally available IBSL-based climate change learning intervention which was developed by a cross disciplinary group of stakeholders including teachers, educational psychologists, instructional designers, and pedagogical experts. This study was part of a national assessment that was designed to assess the educational value of the intervention, and this study describes the results for learning outcomes. Four teachers from different regions of the country (one teacher from each school) agreed to use the intervention and participate in the assessment of the intervention. The intervention consisted of six lessons which each lasted approximately 50 min and included three assessment time points, a prequestionnaire (at the beginning of the intervention), an immediate posttest (immediately after Lesson 2: the HMD/2D video virtual field trip) and a delayed posttest (after the entire intervention, approximately three weeks after experiencing the virtual field trip). Figure 3 provides an overview of the experimental procedure. Teachers were responsible for conducting all six lessons and were instructed to follow a manual with detailed descriptions of the lessons.

The six lessons were structured as follows. The first lesson consisted of a prequestionnaire, and the introduction to a fake news article. Students started by responding to the prequestionnaire, which included questions regarding gender, age, and grade-level. Finally, students read a fake news article followed by a plenary discussion highlighting a controversy around climate change. Lesson 2 consisted of an instruction of the scientific method and a virtual field trip to Greenland. The research team was present during this lesson and randomly assigned students to one of two experimental conditions using randomized ID numbers. The randomized ID number was used to place students to a working group of 3 to 4 students within their assigned condition, which they worked in from lessons 3 to 6. Students were then separated into two separate rooms based on their assigned condition, where they either experienced a virtual field trip to Greenland as a video on a projector screen in the classroom **(**2D video condition: *n* = 53) or as a 360° experience in a HDM (HMD condition: *n* = 49). Following the virtual field trip students took the immediate post-test. Students also completed survey items to measure presence, interest, and enjoyment. These two lessons were conducted on the first day of the study in all schools as can be seen in Fig. 3.

The remaining lessons (lesson 3–6) were designed based on IBSL and generative activity principles of multimedia learning (Fiorella & Mayer, 2021a) and were run by the teachers according to their own schedules so there was variation in the amount of time that passed between each lesson. In lesson 3, Research Design, students generated hypotheses and constructed an experimental design to explain the drivers of climate change in their research groups. Lesson 4, The Experiment, consisted of students developing an idea for an experiment that they could use to test a scientific fact related to climate change. Lesson 5, Interpretation of Results, consisted of students conducting their chosen experiment and interpreting the results. Lastly, lesson 6, Presentation to UN Climate Panel, consisted of research groups presenting their results to a fictitious climate panel made up of their classmates and teacher. This was followed by a delayed posttest that included the same test items as in the immediate posttest.

In summary, lessons 1 and 2 consisted of providing students with fundamental knowledge about concepts such as the greenhouse effect and albedo effect which was also the knowledge assessed in the post-test. Lessons 3–6 consisted of applying that knowledge to develop a research design, experiment, interpret results and present the final project within the IBSL framework. However, no new knowledge was actively provided during sessions 3–6. Thus, students' performance on the delayed posttest in lesson 6 was based on the knowledge gained in lessons 1 and 2, which they were able to apply in lessons 3–6.

The average length of the entire intervention was 19.44 days (SD = 9.87); however, there were differences based on dissimilar schedules across schools (School 1: 8.99 days (31 students); School 2: 13.17 days (20 students); School 3: 22.39 days (25 students); School 4: 33.89 days (26 students). See Appendix Table 3 for a breakdown of the number of students per condition in each school. The experiment followed national and international guidelines for research with human subjects and received approval from the institutional ethics committee.

### Statistical Analyses

Hypotheses 1 through 5 were investigated using independent samples t-tests with presence rating (Hypothesis 1), enjoyment rating (Hypothesis 2), interest rating (Hypothesis 3), immediate posttest score (Hypothesis 4), or delayed posttest score (Hypothesis 5) as the dependent variables, and treatment condition (3D HMD or 2D video) as the independent variable. Furthermore, we investigated the cognitive affective model of immersive learning presented in Fig. 1 using structural equation modeling (SEM). Several statistics were used to assess fit including the Comparative Fit Index (CFI), Tucker Lewis Index (TLI), Root Mean Square Error of Approximation (RMSEA), and Standardized Root Mean Square Residual (SRMR). For both CFI and TLI, acceptable fit values are above 0.95 (Hu & Bentler, 1999; Kline, 2011). For the RMSEA and SRMR acceptable values are below 0.06 and 0.08 respectively (Hu & Bentler, 1999). We conducted the SEM analyses in the R statistical programming language with the standard maximum likelihood (ML) estimation procedure using the Lavaan package.

## Results

First, we investigated whether there were differences between the HMD and 2D groups on demographic characteristics. The HMD and 2D video groups did not differ significantly on mean age (*M* = 14.12, *SD* = 0.67; *M* = 14.15, *SD* = 0.69 respectively), or mean grade level (*M* = 8.18, *SD* = 0.39; *M* = 8.21, *SD* = 0.41 respectively). A chi-square test showed that the HMD (18 males/31 females) and 2D video (21 males/32 females) groups did not differ significantly in terms of the proportion of boys and girls, *X*^2^ (*N* = 102) = 0.090, *p* = 0.764. Furthermore, we calculated the correlation between the change in learning from the immediate test to follow-up test and the time between the two measurements to investigate if the amount of time between the lessons influenced learning. The correlation of *r* = 0.047 (*p* = 0.640) suggests that the time between the two assessments did not influence the results, so the data was grouped together in conducting further analyses.

### Do the Groups Differ on Ratings of for Presence, Enjoyment, and Interest?

We predicted that the HMD group would produce higher presence (Hypothesis 1), enjoyment (Hypothesis 2), and interest (Hypothesis 3) ratings than the video group. These hypotheses were assessed using independent samples t-tests. As shown in the top row of Table 1, the HMD group reported (*M* = 4.18, *SD* = 0.74) significantly higher presence than the video group (*M* = 2.91, *SD* = 1.01), *t*_(100)_ = 7.171, *p* < 0.001, d = 1.43, which supports hypothesis 1. The next row of Table 1 shows that the HMD group (*M* = 4.65, *SD* = 0.63) reported significantly higher enjoyment than the video group (*M* = 3.58, *SD* = 1.22), *t*_(100)_ = 7.171, *p* < 0.001, *d* = 1.10, which supports hypothesis 2. Finally, the next row of Table 1 shows that the HMD group (*M* = 4.06, *SD* = 0.76) reported significantly higher presence than the video group (*M* = 3.45, *SD* = 1.31), *t*_(100)_ = 2.856, *p* = 0.003, *d* = 0.57, which supports hypothesis 3. Overall, these results support hypotheses 1, 2, and 3 and are consistent with the immersion principle, which states that immersion has an impact on affective processing in the learner.

**Table 1:** Mean and standard deviation for the 3D HMD and 2D video groups on the immediate and delayed posttest

Measure	Group				Sig	$d$
	HMD		2D Video			
	M	SD	M	SD		
Presence	4.18 (.74)		2.91 (1.01)		*p* < .001	1.43
Enjoyment	4.65 (.63)		3.58 (1.22)		*p* < .001	1.10
Interest	4.06 (.76)		3.45 (1.31)		*p* = .003	.57
Immediate posttest	18.29 (4.04)		15.57 (4.83)		*p* = .001	.61
Delayed posttest	18.90 (4.35)		15.64 (4.97)		*p* < .001	.70

### Do the Groups Differ on the Immediate Posttest?

An independent samples *t*-test was used to test hypothesis 4 that the HMD group will score higher than the video group on the immediate posttest test in line with the immersion principle. As shown in fourth row of Table 1, the HMD group (*M* = 18.29, *SD* = 4.04) scored significantly higher than the video group (*M* = 15.56, *SD* = 4.82), *t*_(100)_ = 3.072, *p* = 0.001, *d* = 0.61. We therefore conclude that there were significant differences between the HMD and the video conditions on declarative knowledge immediately after experiencing the virtual field trip which is consistent with Hypothesis 4 and the immersion principle from which it is derived.

### Do the Groups Differ on the Delayed Posttest?

An independent samples *t*-test was used to test Hypothesis 5 that the HMD group will score higher on the delayed posttest than the video group in line with the immersion principle. As shown in the bottom row of Table 1, the HMD group (*M* = 18.90, *SD* = 4.35) scored significantly higher than the video group (*M* = 15.64, *SD* = 4.97) on the delayed posttest, *t*_(100)_ = 3.507, *p* < 0.001, *d* = 0.70. Therefore, hypothesis 5 was supported. In general, the results support hypotheses 1 through 5 and indicate that there was a medium effect size difference between the HMD and video conditions on interest (*d* = 0.57), as well as immediate retention (*d* = 0.61), and long-term retention (*d* = 0.70), and a large effect size difference on presence (*d* = 1.43) and enjoyment (*d* = 1.10).

### What Are the Mechanisms of Change by Which Immersion Can Influence Immediate and Delayed Posttest Scores?

**Fig. 4:**
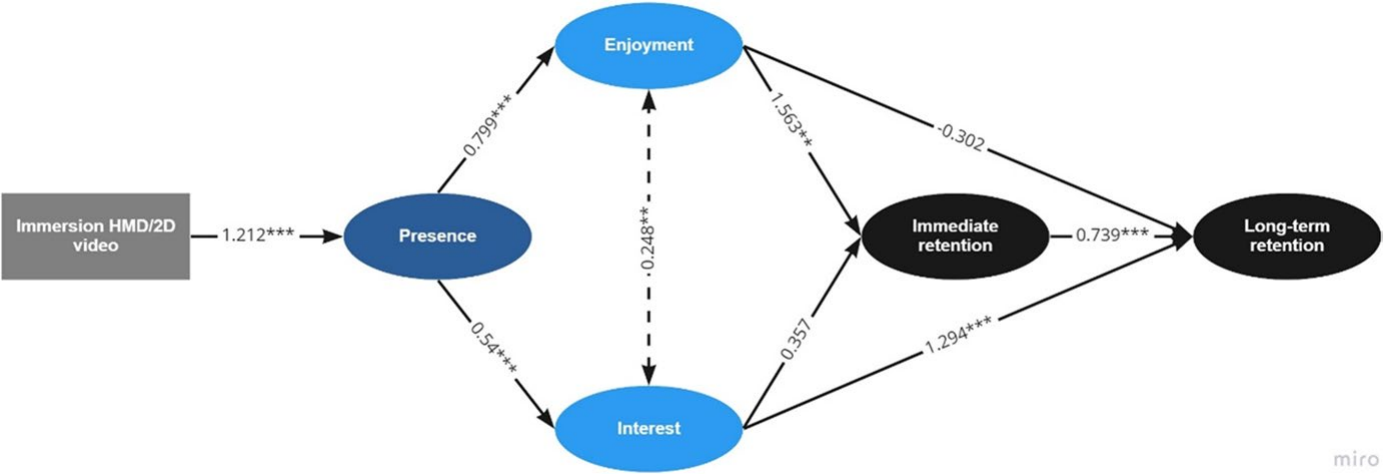
Final model

We investigated whether the link between level of immersion and posttest scores is mediated by presence, interest, and enjoyment as depicted in the cognitive affective model of immersive learning in Fig. 1 using SEM. The results revealed acceptable fit statistics for the CFI = 0.973, and TLI = 0.965 being above the conventional cut-offs, and the SRMR = 0.046 being below the conventional cut-off, however the RMSEA = 0.070 was above the conventional cut-off of 0.60. Figure 4 illustrates the model with the standardized path coefficients. The path from condition to presence was significant (*β* = 1.212, *p* < 0.001, *se* = 0.172), indicating that the students in the HMD condition reported higher presence than those in the video condition as expected. Furthermore, presence was a significant antecedent of enjoyment (*β* = 0.799, *p* < 0.001, *se* = 0.093), and interest (*β* = 0.540, *p* < 0.001, *se* = 0.107) as predicted in the model. The correlation between enjoyment and interest was also significant (*β* = 0.248, *p* = 0.005, *se* = 0.088). The path from enjoyment to immediate posttest score was also significant (*β* = 1.563, *p* = 0.002, *se* = 0.493), but the path from interest to immediate posttest score (*β* = 0.357, *p* = 0.452, *se* = 0.474) was not significant. Therefore, the SEM shows how immediate posttest score is significantly related to enjoyment, but not interest.

Finally, the model illustrates a significant path from interest to delayed posttest score (*β* = 1.294, *p* < 0.001, *se* = 0.340), but the path from enjoyment to delayed posttest score was not significant (*β* = -0.302, *p* = 0.409, *se* = 0.366). The immediate posttest score was also a significant antecedent to delayed posttest score (*β* = 0.739, *p* < 0.001, *se* = 0.071).

Overall, these results present two pathways from instructional media to posttest scores—the enjoyment pathway and the interest pathway. In the enjoyment pathway, immersion influences presence, which is related to enjoyment, which in turn is related to immediate posttest score. In the interest pathway, immersion influences presence, which is related to interest, which in turn is related to delayed posttest score. It appears that enjoyment and interest are involved in learning but in different ways. Enjoyment directly mediates the results on the immediate posttest but not the delayed posttest. Alternatively, interest directly mediates the results on the delayed posttest test but does not the immediate posttest.

## Discussion

### Empirical and Theoretical Contributions

The main finding is that students who learned about environmental science by taking a virtual field trip in HMD (i.e., higher-immersion medium) scored higher on an immediate posttest and a delayed posttest than students taking a virtual field trip presented as onscreen video (i.e., lower-immersion medium). In addition, the HMD group produced higher ratings of presence, interest, and enjoyment than the video group. Overall, these findings support the immersion principle in multimedia learning (Makransky, n.d.; Mayer, 2021a). This is consistent with the results from a recent meta-analysis by Wu et al., (2020) that found a small effects size advantage in favor of immersive VR compared to other traditional means of instruction.

The results of the structural equation model that tested the cognitive affective model of immersive learning in this study provide an empirical explanation for these finding. The SEM showed that the effects of immersion in test performance are mediated by presence, interest, and enjoyment, thereby highlighting the role of affective processes in multimedia learning. This study supports the CAMIL (Makransky & Petersen, 2021) by showing that learning experiences with higher immersion create higher levels of presence, interest, and long-term test performance. Importantly, this study provides evidence for two pathways within the cognitive affective model of immersive learning—the enjoyment pathway and the interest pathway. Theoretically, the findings related to these two pathways supports the call to look beyond global positive emotion in understanding learning processes (Frederickson, 2001). While enjoyment signals pleasure and satisfaction related to a learning activity or achievement, interest motivates exploration and information seeking (Ainley & Hidi, 2014). Therefore, when interest is triggered by a novel event such as the virtual field trip to Greenland, exploratory behavior can occur. Future appraisals of objects in the same domain can then trigger both interest and enjoyment; interest as the alertness and concentration needed for further exploration, and enjoyment as the anticipation of similar knowledge acquisition and successful performance (Ainley & Hidi, 2014).

In the enjoyment pathway, learning with higher levels of immersion leads to higher levels of presence, which is related to higher levels of enjoyment, which, in turn is related to higher levels of performance on an immediate test, which is related to higher levels of performance on a delayed test. This pathway is consistent with the idea that learning through a HMD can cause students to enjoy the experience, leading to performance on an immediate assessment, which in turn leads to better performance on a delayed assessment of the material in the module. The finding is consistent with strong evidence of the relation between enjoyment and learning (Pekrun et al., 2002, 2011). Social agency theory provides a theoretical explanation for how higher presence engages students to invest more cognitive effort to understand material, thereby leading to deeper cognitive processing (Mayer, 2014). The results from the SEM highlight how enjoyment predicted immediate retention but did not directly impact the follow-up retention test. Positive affect experiences such as enjoyment have previously been found to predict on-task cognitive activity but may not necessarily predict long-term achievement (Buff et al., 2011). In the current context, enjoyment may not follow past lesson 2 as the following lessons were not immersive and may have been considered business as usual by students, whereas the IBSL interventions fostered further exploration activities, thereby creating a potential environment for maintaining situational interest.

In the interest pathway, learning with higher immersion media leads to higher levels of presence, which is related to higher levels of interest, which, in turn, is related to higher levels of long-term test performance. This pathway is consistent with the idea that learning in HMD can cause students to become more interested in the material, which leads to better learning over the course of an instructional module as manifested in better performance on long-term assessments of learning. Hidi and Renninger (2006) argues that interest is a unique motivational variable because when interest is triggered it can be considered an emotion; however, interest includes both affective and cognitive components as it develops and is maintained. Interest engages the student with the new and puzzling task and contributes to the sustained effort required for achieving satisfying outcomes which can also involve persisting through negative feelings of frustration (Renninger, 2000). Recent reviews (Hidi & Renninger, 2006; Renninger & Hidi, 2011) find a positive association between interest (both situational and individual) and learning outcomes.

The findings support a deeper understanding of how creating unique educational experiences that feel real (i.e., create a high level of presence) through immersive technology can influence learning through different affective and cognitive processes including enjoyment and interest. Together both enjoyment and interest are hallmarks of capable, confident and enthusiastic learners (Ainley & Hidi, 2014; p. 223). The immersion principle of multimedia learning highlights how it is not the technology in itself, but rather appropriate instructional design that matches the affordances of immersive technology that can improve learning. Evidence-based instructional design principles were implemented in the HMD and 2D versions of the virtual field trip and the combination of these principles within an immersive HMD experience resulted in higher levels of presence, enjoyment, interest, and immediate as well as long-term retention.

### Practical Implications

The main purpose of this research has been to systematically test the immersion principle in multimedia learning to understand if and how it works within a realistic middle school science context. We concur with Chandler (2009), who argues that it is important to look beyond the “wow” factor of dynamic visualizations for instruction. By providing stakeholders such as Ana (the middle school geography teacher who was described in the introduction of this paper) with more evidence about the benefits and limitations of using HMD based lessons compared to videos we can ensure that immersive media is used based on actual educational value. The results of the study encourage instructors to incorporate lessons involving HMD experiences into their instructional programs, particularly as an introduction. These experiences can help create interest in the material that leads to better learning engagement and learning outcomes over the course of an instructional module. For example, taking a virtual field trip as an initial step in an instructional module may be a good way to create enjoyment that maintains engagement over the short-term and to instill interest that causes students to engage with the material over the long-term.

An important consideration is that virtual field trips make it possible to experience things that are too expensive, dangerous, or impossible in the real world. Reviews on the use of physical field trips show that field trips serve best as opportunities for exploration, discovery, first-hand and original experiences (DeWitt and Storksdieck, 2008) and that they can increase student interest, knowledge, and motivation (Behrendt & Franklin, 2014). The evidence from this study suggests that HMDs provide teachers with opportunities to take advantage of these factors within the safety of the classroom environment and that the benefits are higher in terms of presence, enjoyment, interest and immediate as well as long-term retention compared to a 2D video.

It is important to highlight that there are also practical challenges to using HDMs in realistic educational contexts and many other factors play a role in teachers' choices to use novel technology in the classroom. Bower and colleagues (2020) investigated preservice teachers’ perceptions of IVR and their behavior intentions to use the technology. They found that hedonic motivation (i.e., enjoyment) was the most important factor for intentions to use the technology, but also report that intentions to use IVR were constrained by external barriers such as access, logistics, support; internal barriers such as attitudes and experience; and design issues such as technical skills, and ideas for pedagogically meaningful tasks.

In practice the decision to use a HMD or a 2D video in an educational activity will depend on a number of different factors that go beyond the educational value of the different media. In this study we found that the HMD resulted in a large effect size advantage over the 2D video on the outcomes of presence (*d* = 1.43), and enjoyment (*d* = 1.10), and medium effect size advantage on the outcomes of interest (*d* = 0.57), immediate retention (*d* = 0.61), and delayed retention (*d* = 0.70). Although this provides evidence for the educational value of immersive technology, stakeholders such as Ana will also have to balance other important factors such as the cost of purchasing and maintaining equipment, time constraints, practical considerations such as how to administer a HMD experience to an entire class, as well as privacy and safety issues that have all been highlighted as limitations when using VR in the classroom (Pimentel et al., in press). Although initial investment in using immersive technology can come at the cost of other investments, balancing these different considerations will depend greatly on different contextual factors. These factors will change rapidly in line with technological and societal developments. Current examples include the need to rethink education during the COVID-19 pandemic and the anticipation of the Metaverse. In general, it is likely that it is not a matter of whether stakeholders like Ana will implement immersive technology in their lesson, but rather a matter of when and how this will be done. Our sincere hope is that the development and implementation of immersive lessons in education is conducted based on research evidence about how these experiences can influence learning.

## Limitations and Future Directions

This was a media comparison experiment in which we compared learning academic content with one medium versus another. Media comparison studies are subject to methodological criticism based on the claim that it is difficult to maintain experimental control in which the two groups receive identical instructional content and instructional methods (Clark, 2001; Mayer, 2014). In the present study, the verbal content was identical in the two groups and the visual content was identical in the groups, except that it was either viewed in 3D using a HMD or in 2D through a video. The instructional method was identical except that students in the video group could not interact with the material whereas students in the HMD could change their point of view by turning their heads to view different parts of the virtual environment. In the present study, interactivity is an inherent component of the HMD medium but not the video medium, so it is not possible to determine whether the effects are attributable to immersion or interactivity. Future research is needed to disentangle to the roles of immersion and interactivity, perhaps by adding interactivity to a video lesson through a mouse or touchpad.

Although the virtual field trip was a short intervention lasting only 9 min and 46 s, the finding that there were differences between groups on the five dependent variables, and that a difference on the delayed posttest was identified almost 3 weeks after the virtual field trip shows that even short virtual field trip experiences can have an impact on long-term outcomes due to creating a greater interest for the topic. However, future studies should investigate the value of using longer and different types of virtual field trips. Future research should also investigate if the findings generalize to different populations including different age groups as the meta-analysis by Wu et al., (2020) found that the advantages of immersive lessons were higher in K-12 compared to post-secondary contexts. Geographical location may also be relevant for the topic of climate change. The current study took place in a European country that is not currently experiencing negative consequences due to climate change, and the relevance of such a lesson may be higher for students who directly experience impacts.

A limitation in this study was the relatively small sample size. The small sample size could be problematic for the stability of the SEM model, so the conclusions should be interpreted with caution. The study was a national evaluation of the value of using HMDs in education, which allowed us to use an actual educational intervention within a classroom setting. However, future studies should investigate the longitudinal value of using HMDs compared to videos with larger sample sizes. Another limitation in this study is related to the measurement instruments that were used. The interest measure did not differentiate between situational and individual interest (Renninger & Hidi, 2016), which could be informative in investigating the short- and long-term impact of immersive lessons.

A further consideration is that students in both conditions participated in a number of lessons (i.e., lessons 3 to 6) between the immediate and delayed posttest. This was important for the design of the study because the immersion principle would suggest that students in the HMD condition would exert more effort in those lessons based on having more situational interest than students in the video condition. It was not possible to measure effort during these lessons in the current context, but future research should attempt to obtain process measures to further investigate this hypothesis.

In the current study the video condition was administered as a collective session while the HMD condition was administered as an individual session. This decision was made to maintain ecological validity of how the media would be used in a classroom. More specifically, the teachers determined that giving each student access to the video on their own device would cause more distractions than using a collective video. Regardless of how a video is presented there is the possibility that students communicate and disturb each other; however, observations of the session indicated that this was not a great problem as students generally were very focused on watching the video in this experiment. One affordance of using HMDs is that teachers are able to immerse students in the lesson, making it impossible to communicate and disturb each other, which is not possible with a video session. Nevertheless, future research should compare using a video or HMD in individual sessions.

Although we included enjoyment and interest in the SEM; many other factors influence the process of learning in immersive lessons. Such factors include interactivity, cognitive load, self-regulation, and particular individual differences variables such as spatial skills, which should be included in future research. Future research could also investigate other media as a comparison condition to HMD including real field trips, books etc., as the results of the current study are focused on differences between experiencing a virtual field trip in HMD compared to a 2D video.

In conclusion, we have found that a virtual field trip experienced through a HMD is superior to the same virtual field trip when presented as a 2D video on the outcomes of presence, enjoyment, interest, as well as short-term and long-term retention in the context of a middle school IBSL climate change intervention. The SEM model showed that enjoyment directly mediates the results on the immediate posttest but not the delayed posttest. Alternatively, interest directly mediates the results on the delayed posttest test but does not the immediate posttest. The research provides additional support of the immersion principle in multimedia learning and initial findings that offer a better idea of how immersive lessons can influence long-term learning outcomes. We encourage future research to further test the cognitive affective model of immersive learning presented in this article and add to the model to eventually gain a stronger understanding of the variables and processes that play a role in learning with immersive technology.

## Appendix

**Table 2:** Measures

Immediate Posttest, and Delayed Posttest	Source
How much ice is melting in Greenland yearly compared to previously? a) Twice as much, **b) Three times as much**, c) Four times as much, d) Five times as much	Developed for the study. Maximum score = 27; (Cronbach’s alpha = 0.78, 0.78 in the immediate and follow-up tests respectively)
What is meant by the greenhouse effect? **a) An isolating layer of gasses around the Earth, which keeps in the heat,** b) Air pollution from factories and cars, which creates a “duvet” over many cities, c) An isolating layer of gasses around the Earth, which keeps Earth cool, d) When a hole is made in the ozone layer and Earth’s oxygen is released into the atmosphere	
Which gas, which contributes to an increase in the greenhouse effect, has been released into the atmosphere in large quantities during the last few years? a) Oxygen, b) Carbon monoxide, **c) Carbon dioxide**	
When did the manmade augmentation of the greenhouse effect really spark off? **a) During the industrial revolution in the nineteenth century,** b) During the oil crisis in the 1970’s, c) During the Second World War in the 1940’s, d) During the climate crisis in the 1990’s	
The biggest human contribution to the greenhouse effect stems from a) Plastic in the oceans, **b) Fossil fuels**, c) Nuclear power, d) Solar and wind energy	
What has been experimented with in order to counteract the acidification of the oceans around Greenland? a) Banning the discharge of meltwater (True/**False**), b) Removing plastic and other pollutants from the oceans (True/**False**), Establishing forests of seaweed (**True**/False), c) Adding a basic material to the ocean (True/**False**)	
What is true and false about albedo? a) Albedo is a layer of gasses that regulate Earth’s temperature (True/**False**), b) Albedo is a measure of the radiation that is reflected back into space (**True**/False), c) Albedo is also the name of an international agreement concerning global warming (True/**False**), d) Albedo is a measure of the radiation that is absorbed when sun hits the surface of the Earth (**True**/False), e) O2, CO2, NO2, and CNO2 have high albedos (True/**False**), f) Seawater and land have high albedos (True/**False**), g) Snow and ice have high albedos (**True**/False)	
What happens to the planet when the ice and snow in Greenland melt? a) The temperature decreases because the ice and snow release cooling gasses (True/**False**), b) The temperature decreases because the oceans are cooled (True/**False**), c) The air temperature increases because there is less snow and ice (**True**/False), d) It leads to a higher concentration of corals (True/**False**), e) It leads to an increased reflection of energy, which cools the planet via ice-albedo feedback (True/**False**)	
What happens if soot is covering ice and snow? a) The soot isolates and prevents the ice and snow from melting (True/**False**), b) The soot is gradually dissolved and nothing else happens (True/**False**), c) The soot is cooled and releases greenhouse gasses (True/**False**), d) The soot absorbs the radiation of the sun resulting in the snow and ice melting (**True**/False)	
What connection is there between the greenhouse effect and the melting of ice? a) There is no connection, **b) When water goes from solid to liquid form, CO2 is released**, c) When ice melts, sea temperatures increase—this makes plastic in the oceans release greenhouse gasses, d) When water goes from solid to liquid form, CO2 is absorbed	
How many people is expected to require evacuation during this century as a consequence of rising sea levels produced by melting ice? **a) Between 10 million and a 100 million**, b) Approximately 5 million, c) Approximately 500.000, d) Less than 100.000	
**Presence Survey**	Adapted from Makransky et al., (2017) Cronbach alpha = .94
The virtual field trip to Greenland seemed real to me	
The virtual field trip to Greenland gave me the feeling of being there myself	
My experience in the virtual environment seemed as though I was there in the real world	
While I was in the virtual environment, I had a sense of “being there”	
I was completely captivated by the virtual world	
**Interest Survey**	Adapted from Thisgaard and Makransky, (2017) Cronbach alpha = .95
I am interested in the scientific explanations for climate change	
I am interested in the albedo effect	
I am interested in the greenhouse effect	
I am interested in climate change	
**Enjoyment Survey**	Adapted from Tokel and İsler (2015) Cronbach alpha = .95
I like to learn about climate change through VR/video	
I think that it is fun to use VR/video to learn about climate change	

All of the items were administered in the local language and have been translated in this table.

All items in the presence, interest, and enjoyment surveys were answered on a five-point Likert scale with the following anchors: 1 = strongly disagree; 2 = disagree; 3 = neither agree nor disagree; 4 = agree; 5 = strongly agree.

**Table 3:** Overview of number of students in each condition by school

	Video	VR
School 1 (2 classes)	13	18
School 2 (1 class)	11	9
School 3 (2 classes)	13	12
School 4 (2 classes)	16	10
Total	53	49
